# Physics-based early warning signal shows that AMOC is on tipping course

**DOI:** 10.1126/sciadv.adk1189

**Published:** 2024-02-09

**Authors:** René M. van Westen, Michael Kliphuis, Henk A. Dijkstra

**Affiliations:** Institute for Marine and Atmospheric Research Utrecht, Utrecht University, Princetonplein 5, Utrecht 3584 CC, Netherlands.

## Abstract

One of the most prominent climate tipping elements is the Atlantic meridional overturning circulation (AMOC), which can potentially collapse because of the input of fresh water in the North Atlantic. Although AMOC collapses have been induced in complex global climate models by strong freshwater forcing, the processes of an AMOC tipping event have so far not been investigated. Here, we show results of the first tipping event in the Community Earth System Model, including the large climate impacts of the collapse. Using these results, we develop a physics-based and observable early warning signal of AMOC tipping: the minimum of the AMOC-induced freshwater transport at the southern boundary of the Atlantic. Reanalysis products indicate that the present-day AMOC is on route to tipping. The early warning signal is a useful alternative to classical statistical ones, which, when applied to our simulated tipping event, turn out to be sensitive to the analyzed time interval before tipping.

## Teaser

A complex global climate model shows an AMOC tipping event.

## INTRODUCTION

The Atlantic meridional overturning circulation (AMOC) effectively transports heat and salt through the global ocean (*1*) and strongly modulates regional and global climate. Continuous section measurements of the AMOC, available since 2004 at 26°N from the RAPID-MOCHA array (*2*), have shown that the AMOC strength has decreased by a few Sverdrups (1 Sv = 10^6^ m^3^ s^−1^) from 2004 to 2012, and thereafter, it has strengthened (*3*) again. Longer timescale variability of the AMOC strength, estimated by using sea surface temperature (SST) time series based on “fingerprint” patterns (*4*), indicates that the AMOC weakened by 3 ± 1 Sv since about 1950. From proxy records, it has been suggested that the AMOC is currently in its weakest state in over a millennium (*5*).

The AMOC has been labeled as one of the tipping elements in the climate system (*6*, *7*), indicating that it may undergo a relatively rapid change under a slowly developing forcing. The AMOC is particularly sensitive to the ocean’s freshwater forcing, either through the surface freshwater flux (e.g., precipitation) or by input of fresh water due to river runoff or ice melt (e.g., from the Greenland Ice Sheet). Although no AMOC tipping has been found in historical observations, there is much evidence from proxy records that abrupt AMOC changes have occurred in the geological past during the so-called Dansgaard-Oeschger events (*8*–*10*).

Classical early warning indicators, such as the increase in the variance and/or the (lag-1) autocorrelation, when applied to SST-based time series, suggest that the present-day AMOC approaches a tipping point before the end of this century (*11*, *12*). Apart from the fact that the SST-based AMOC fingerprints may not represent the AMOC behavior adequately, many (statistical) assumptions are required to estimate the approaching AMOC tipping point (*12*–*15*). Hence, there is strong need for a more physics-based, observable, and reliable early warning indicator that characterizes the AMOC tipping point.

## RESULTS

### AMOC collapse

To develop such an early warning indicator, we performed a targeted simulation to find an AMOC tipping event in the Community Earth System Model (CESM; version 1.0.5). This CESM version, which has been used in the Coupled Model Intercomparison Project (CMIP), phase 5, has horizontal resolutions of 1° for the ocean/sea ice and 2° for the atmosphere/land components (see Materials and Methods).

**Fig. 1.**
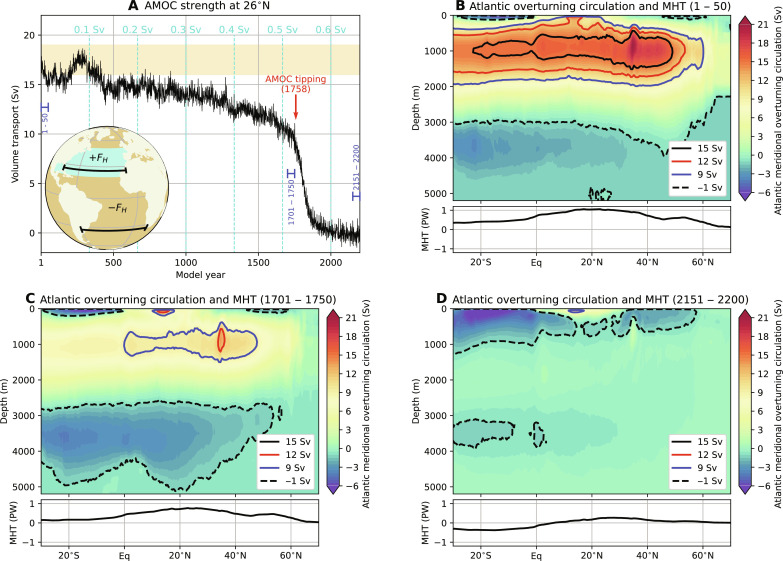
AMOC collapse. (**A**) The AMOC strength at 1000 m and 26°N, where the yellow shading indicates observed ranges (*60*, *61*). The cyan-colored lines indicate the magnitude of *F*_H_. The red arrow indicates the AMOC tipping point (model year 1758; fig. S1, A and B), and the blue sections indicate the 50-year periods used in (B) to (D). Inset: The hosing experiment where fresh water is added to the ocean surface between 20°N and 50°N in the Atlantic Ocean (+*F*_H_) and is compensated over the remaining ocean surface (−*F*_H_). The black sections indicate the 26°N and 34°S latitudes over which the AMOC strength and freshwater transport (*F*_ovS_) are determined, respectively. (**B** to **D**) AMOC streamfunction (Ψ) and Atlantic meridional heat transport (MHT; see also fig. S2) for model years 1 to 50, 1701 to 1750, and 2151 to 2200. The contours indicate the isolines of Ψ for different values.

We start from a statistical equilibrium solution of a preindustrial control simulation (*16*) and keep greenhouse gas and solar and aerosol forcings constant to preindustrial levels during the simulation. A quasi-equilibrium approach (*17*–*19*) is followed by adding a slowly varying freshwater flux anomaly *F*_H_ in the North Atlantic over the region between latitudes 20°N and 50°N. This freshwater flux anomaly is compensated over the rest of the domain, as shown in the inset of Fig. 1A. We linearly increased the freshwater flux forcing with a rate of 3 × 10^−4^ Sv year^−1^ until model year 2200, where a maximum of *F*_H_ = 0.66 Sv is reached. Such a simulation has not been conducted before with a complex global climate model (GCM) (i.e., used in CMIP5 and beyond) as the CESM version used here because of the high computational costs and it cannot easily be repeated for a suite of different GCMs.

Under increasing freshwater forcing, we find a gradual decrease (Fig. 1A) in the AMOC strength (see Materials and Methods). Natural variability dominates the AMOC strength in the first 400 years; however, after model year 800, a clear negative trend appears because of the increasing freshwater forcing. Then, after 1750 years of model integration, we find an abrupt AMOC collapse (fig. S1, A and B). The AMOC strength is about 10 Sv in model year 1750 and decreases to 2 Sv 100 years later (model year 1850) and eventually becomes slightly negative after model year 2000. Such a transient AMOC response (model years 1750 to 1850) is spectacular considering the slow change in the freshwater forcing (i.e., ∆*F*_H_ = 0.03 Sv). The characteristic meridional overturning circulation and associated northward heat transport in the Atlantic Ocean have decreased to nearly zero and by 75% (at 26°N), respectively, after model year 2000 (Fig. 1, B to D, and fig. S2A).

This result differs substantially from earlier model simulations with GCMs that have used extremely large freshwater forcing [e.g., 1 Sv per year over 50°N to 70°N (*20*)] or large initial salinity perturbations (*21*). The AMOC collapse in these simulations is a direct response to the very strong forcing, whereas in our model simulations, which are more akin to the simulations in Earth System Models of Intermediate Complexity (*17*, *18*), the collapse is primarily a response due to internal feedbacks. This can be quantified by looking at the AMOC change per cumulative change in North Atlantic freshwater forcing, which is about $R=\frac{8\;\text{Sv}}{1.5\;\text{Sv}\;\text{year}}=5.3\;{\text{year}}^{-1}$ in our hosing simulations (model years 1750 to 1850) and about $R=\frac{18\;\text{Sv}}{50\;\text{Sv}\;\text{year}}=0.36\;{\text{year}}^{-1}$ for the strong forcing of 1 Sv/year over a 50-year period for the results in (*20*). For *R* ≪ 1 year^−1^, the AMOC changes are primarily driven by the freshwater forcing, and for *R* ≫ 1 year^−1^, the AMOC changes are mainly induced by internal feedbacks. Also, on the basis of the change in the AMOC per forcing change (here about 8-Sv AMOC change due to a forcing change of 0.03 Sv), it is clear that we found an AMOC tipping event (*6*) in the CESM simulation, which is the first one found in a complex GCM. The FAMOUS model (*18*), forced under a slowly varying freshwater forcing of 5 × 10^−4^ Sv year^−1^, shows AMOC tipping, and there, the AMOC change per cumulative freshwater forcing is $R=\frac{17\;\text{Sv}}{17\;\text{Sv}\;\text{year}}=1\;{\text{year}}^{-1}$ . This value of *R* indicates that both freshwater forcing and internal feedbacks are both important in inducing AMOC changes. This is a factor 5 smaller with respect to the CESM, which is likely related to the coarser horizontal ocean resolution (2.5° × 3.75°) and associated higher viscosity in FAMOUS.

The differences in important ocean observables between the two different AMOC states (averages over model years 2151 to 2200 minus years 1 to 50) are presented in fig. S3. Figure S3A shows a cooling of the Northern Hemispheric SSTs when the AMOC collapses, with SST differences as large as 10°C near western Europe. On the contrary, the SSTs in the Southern Hemisphere increase because of the collapse resulting in a distinct seesaw pattern between the hemispheres (*22*). This pattern arises from the reduced meridional heat exchange between the hemispheres (fig. S2). The North Atlantic upper 100-m salinities are also strongly influenced under the AMOC collapse (fig. S3B). Note that salinities outside of the Atlantic have increased partly because of the freshwater flux compensation used in the setup of the quasi-equilibrium experiment. From the changes in the annual maximum mixed-layer depth (fig. S3C), it can be deduced that deep convection ceases in the North Atlantic (around Greenland), which is in accordance with the reversed AMOC state (Fig. 1D). Other regions, such as the Southern Ocean, show an increase in the mixed-layer depth. The weakening of the AMOC results, via geostrophic balance, in dynamic sea-level rise in the Atlantic Ocean (fig. S3D) and some coastal regions experiences more than 70 cm of dynamic sea-level rise.

### Climate impacts

The SST changes due to AMOC collapse also affect the atmosphere and global sea-ice distribution. The atmospheric responses (fig. S4) consist of a seesaw pattern in the 2-m surface temperature, a southward intertropical convergence zone (ITCZ) shift, and the strengthening of the Hadley Cell in the Northern Hemisphere. The stronger meridional temperature gradient over the Northern Hemisphere amplifies the subtropical jet, while the opposite happens in the Southern Hemisphere. During the gradual AMOC weakening over the first 1400 model years, there were no significant trends [*P* > 0.05, two-sided *t* test (*23*)] in the global mean surface temperature or in the global sea-ice area. Under the AMOC collapse, the Arctic (March) sea-ice pack extends down to 50°N and there is a gradual retreat of the Antarctic (September) sea-ice pack (fig. S5). The vast expansion of the Northern Hemispheric sea-ice pack amplifies further Northern Hemispheric cooling via the ice-albedo feedback. These findings are qualitatively similar to those in (*20*), in which AMOC is strongly weakened to 3 to 4 Sv.

**Fig. 2.**
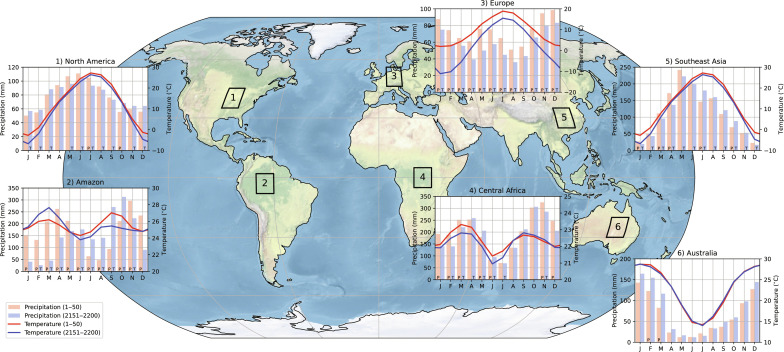
Climograph for different regions. The climograph for six different regions (spatial average over the 10° × 10° boxes), where the bars indicate the monthly precipitation and the curves indicate the monthly temperatures. The climograph is determined over model years 1 to 50 (red bars and curves) and model years 2151 to 2200 (blue bars and curves). Note the different vertical ranges for each climograph. The letters P and T in the bars indicate significant (*P* < 0.05, two-sided Welch’s *t* test) monthly differences for precipitation and temperature, respectively.

The aforementioned ocean, atmosphere, and sea-ice responses strongly influence the regional climates across the globe (Fig. 2). The European climate is significantly different after the AMOC collapse, whereas for other regions only specific months undergo significant changes. The Amazon rainforest also shows a drastic change in their precipitation patterns due to ITCZ shifts, and the dry season becomes the wet season and vice versa. These AMOC-induced precipitation changes could severely disrupt the ecosystem of the Amazon rainforest (*7*, *24*, *25*) and potentially lead to cascading tipping (*26*–*28*). The Northern Hemisphere shows cooler temperatures after the AMOC collapse, while the opposite is true for the Southern Hemisphere, although not all changes are significantly different (due to large interannual variability).

**Fig. 3.**
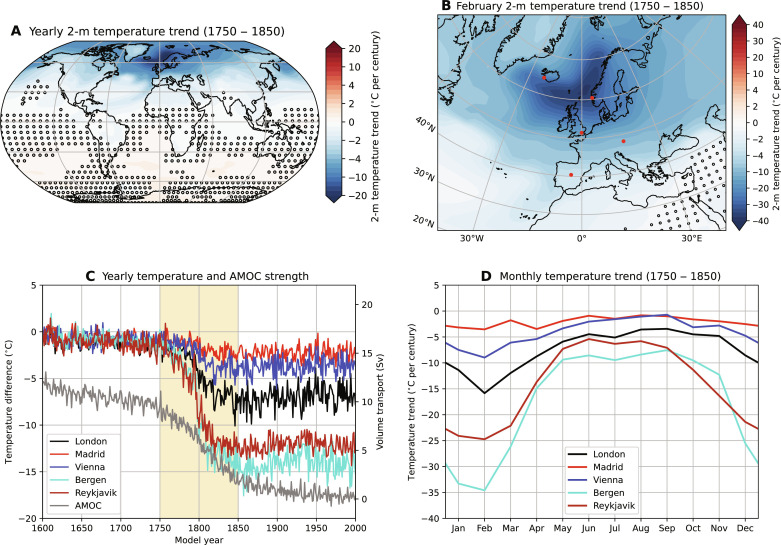
Surface temperature response during AMOC collapse. (**A**) Yearly averaged 2-m surface temperature trend (model years 1750 to 1850). The markers indicate nonsignificant trends [*P* > 0.05, two-sided *t* test (*23*)]. (**B**) Similar to (A) but now for the February 2-m surface temperature trend. The red dots indicate five different cities used in (C) and (D). Note the different color bar ranges between (A) and (B). (**C**) Temperature difference (with respect to model year 1600) for five different cities, including the AMOC strength. The trends are determined over model years 1750 to 1850 (yellow shading) during which the AMOC strength strongly decreases. (**D**) Monthly temperature trends for the five different cities.

The European climate is greatly affected (Fig. 3A) under the AMOC collapse. Note that the corresponding changes occur within a relatively short period (model years 1750 to 1850) and under a very small change in surface freshwater forcing. The yearly averaged atmospheric surface temperature trend exceeds 1°C per decade over a broad region in northwestern Europe, and for several European cities, temperatures are found to drop by 5° to 15°C (Fig. 3C). The trends are even more notable when considering particular months (Fig. 3B). As an example, February temperatures for Bergen (Norway) will drop by about 3.5°C per decade (Fig. 3D). These relatively strong temperature trends are associated with the sea-ice albedo feedback through the vast expansion of the Arctic sea-ice pack (fig. S5A).

### Physics-based early warning indicator

From idealized ocean-climate models, it has been suggested that the freshwater transport of the AMOC at 34°S, indicated by *F*_ovS_ (see Materials and Methods), is an important indicator of AMOC stability (*29*–*33*). The reason is that this quantity is a measure of the salt-advection feedback strength, thought crucial in AMOC tipping. This feedback describes the amplification of a freshwater perturbation in the North Atlantic through a weakening of the AMOC, which leads to less northward salt transport and, hence, amplification of the initial freshwater perturbation (*34*, *35*).

**Fig. 4.**
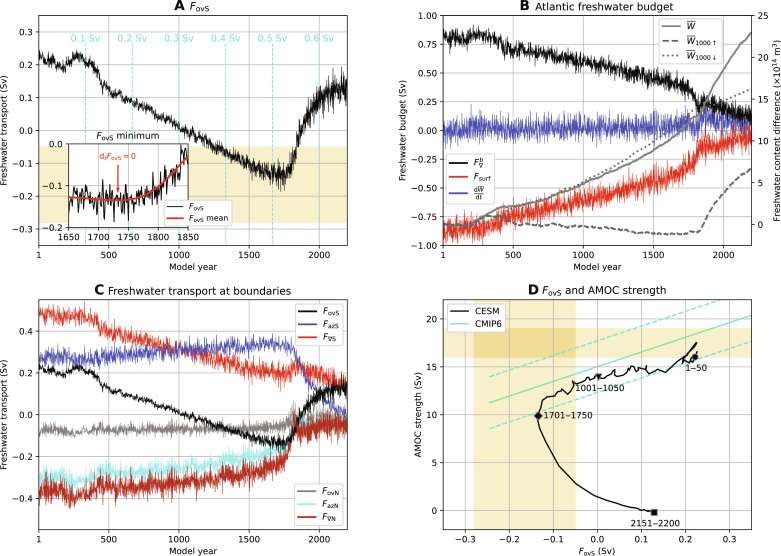
Freshwater transport by the AMOC and Atlantic Ocean freshwater budget. (**A**) Freshwater transport by the AMOC at 34°S, *F*_ovS_. The cyan-colored lines indicate the magnitude of *F*_H_. Inset: Zoom in around *F*_ovS_ minimum (model year 1732; fig. S1, C and D), where the *F*_ovS_ mean is determined from cubic splines (here for 50-year averages, see Materials and Methods). (**B**) Atlantic Ocean freshwater content ($\bar{W}$ ) difference (with respect to time mean over model years 1 to 50) and with the freshwater content difference of the upper 1000 m ($\bar{W}$_1000↑_) and below 1000 m ($\bar{W}$_1000↓_). The freshwater budget components of the freshwater convergence ($F_{\nabla }^{b}$, freshwater transport at 34°S, 60°N, and the Strait of Gibraltar), surface freshwater fluxes (*F*_surf_), and changes in the freshwater content ( $\frac{\mathrm{dW}}{\mathrm{dt}}$). (**C**) Meridional freshwater transport at the Atlantic boundaries of 34°S and 60°N for the overturning component (*F*_ovS_ and *F*_ovN_), the azonal (gyre) component (*F*_azS_ and *F*_azN_), and the total freshwater transport (*F*_∇S_ and *F*_∇N_). Positive (negative) values indicate the northward transport of net fresh water (salinity). (**D**) *F*_ovS_ and AMOC strength over time. The time series are displayed as 25-year averages (to reduce the variability of the time series). The markers indicate the 50-year average over a particular period. The cyan-colored curve indicates the present-day (1994 to 2020) CMIP6 regression and 1 SD (*37*). The yellow shading in (A) and (D) indicates observed ranges (*21*, *42*, *60*, *61*) for *F*_ovS_ and AMOC strength.

In the CESM results here (Fig. 4A), *F*_ovS_ is positive at the beginning of the simulation, which indicates that the AMOC exports net salinity (with respect to reference salinity of 35 g kg^−1^) out of the Atlantic. This is not in agreement with observations (*36*, *37*), which is a well-known bias in CMIP phase 3 (*38*), phase 5 (*21*), and phase 6 (*37*) models. In the CMIP phase 6 (CMIP6) models, this bias is mainly due to large biases (compared to observations) in the freshwater flux over the Indian Ocean (*37*).

The Atlantic Ocean is a net evaporative basin, and as imposed, its surface freshwater flux increases at the same rate as the freshwater forcing before the AMOC collapse (Fig. 4B). A larger salinity transport into (and/or larger freshwater transport export out of) the Atlantic Ocean is needed to balance the Atlantic’s freshwater budget (*29*), resulting in a declining freshwater convergence ($F_{\nabla }^{b}$; Fig. 4B).

The freshwater convergence is not fully compensating the surface freshwater transport changes (≈0.1-Sv difference in model year 1700), resulting in freshwater storage in the Atlantic Ocean ( $\bar{W}$ ; Fig. 4B), in particular, below 1000-m depths. The freshwater convergence changes are primarily driven by *F*_ovS_ changes (82%), followed by (azonal) gyre changes at 60°N (32%) (Fig. 4C). Note that the (azonal) gyre changes at 34°S negatively contribute (−15%) to freshwater convergence changes.

The results in Fig. 4 show that *F*_ovS_ plays a dominant role in balancing the Atlantic’s freshwater budget under the imposed freshwater forcing. Before the AMOC collapse, the salinity changes (at 34°S) are larger than the meridional velocity changes (fig. S6) and indicate that *F*_ovS_ changes are primarily induced by salinity changes. Although the individual contributions of salinity and velocity to *F*_ovS_ changes cannot be quantified, both factors contribute to *F*_ovS_. This is well demonstrated after the AMOC collapse (right column in fig. S6), where the velocity response decreases the (negative) magnitude of *F*_ovS_. When the salinities adjust to the collapsed state, *F*_ovS_ becomes positive again, which is in line with the analyses from idealized climate model studies (*31*, *32*). The range of *F*_ovS_ and AMOC changes in the northward overturning regime (until model year 1750) is within (Fig. 4D) that of present-day simulations of CMIP6 models (*37*). However, the most important result here is that *F*_ovS_ goes through a minimum (inset, Fig. 4A) very close to the AMOC collapse. The *F*_ovS_ minimum is at model year 1732 (1727 to 1740, 10 and 90% percentiles), and the AMOC tipping point, determined from break regression analysis [(*39*); fig. S1], is at model year 1758 (1741 to 1775, 10 and 90% percentiles). Conceptual AMOC models (*29*, *40*) clearly identify such a minimum with a saddle-node bifurcation (*41*), which is the AMOC tipping point in these models.

At the *F*_ovS_ minimum, an increment in the anomalous surface freshwater forcing weakens the AMOC further. The velocity changes are now dominating the *F*_ovS_ response, while salinity changes primarily induce the negative *F*_ovS_ response before the minimum. The weaker meridional velocities decrease the magnitude of *F*_ovS_, and as *F*_ovS_ is negative, this then results in a minimum. Therefore, the weaker AMOC carries then less salinity into the Atlantic Ocean, and the dominant balance between *F*_H_ and *F*_ovS_ changes cannot be sustained. This imbalance also results in the largest Atlantic freshwater content increase of 0.50 × 10^14^ m^3^ (∆$\bar{W}$ between model years 1750 and 1726) during the first 1750 model years, which further destabilizes the AMOC. The tipping point is at slightly higher values of *F*_H_ (and hence slightly later in the simulation) than the value at the *F*_ovS_ minimum, so the latter is a lower bound for tipping. This is because the freshwater budget is not entirely balanced by *F*_ovS_ changes under the freshwater forcing (Fig. 4, B and C). Although these responses under the surface freshwater forcing are fairly small compared to *F*_ovS_ changes, they allow the existence of a near-equilibrium AMOC state for larger *F*_H_ values than that at the *F*_ovS_ minimum.

An alternative explanation for the minimum is that since *F*_ovS_ gets more negative from surface salinification (directly from −*F*_H_) and since an AMOC collapse would cause *F*_ovS_ to go toward zero (i.e., increase), there would be a minimum in *F*_ovS_ at the start of the AMOC collapse. About 70% of the negative *F*_ovS_ response (up to model year 1750) originates from freshwater transport changes in the upper 500 m at 34°S. The freshwater transport changes are dominated by the upper 500-m salinity responses (fig. S6) and can be connected to surface salinification (from −*F*_H_). The North Atlantic Deep Water contributes another 20% to the negative *F*_ovS_ response; the surface waters near the deep water formation regions freshen (through +*F*_H_) and, after deep convection, influence the salinity properties of the North Atlantic Deep Water. This view suggests a passive role of *F*_ovS_ in the AMOC collapse, and the *F*_ovS_ minimum is then expected to coincide with the AMOC collapse. However, the *F*_ovS_ minimum occurs 25 years (9 to 41, 10 and 90% percentiles) before the AMOC tipping event. The variability in *F*_ovS_ also increases toward the *F*_ovS_ minimum, indicating that the AMOC loses resilience and such behavior is typically found when approaching a saddle-node bifurcation. Bifurcation studies with idealized ocean-climate models also indicate that the *F*_ovS_ minimum is found at lower values of the freshwater forcing than the AMOC tipping point (*31*, *32*), which is providing support to the interpretation of the CESM results.

**Fig. 5.**
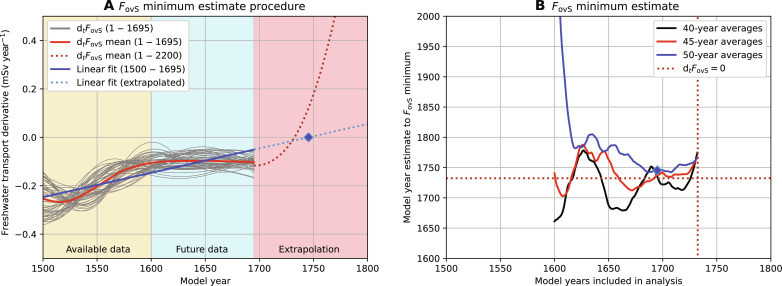
*F*_ovS_ minimum estimate. (**A**) *F*_ovS_ minimum estimate procedure (here for 50-year averages, see Materials and Methods). The time derivative of *F*_ovS_ (d*_t_F*_ovS_, red curve) is determined over the available and future data (up to model year 1695), and then a linear trend is determined (model years 1500 to 1695), which is extrapolated to find the zero (diamond label). (**B**) *F*_ovS_ minimum estimate for varying model years (i.e., available and future data, starting from model year 1500) and different averaging periods. The dotted lines and diamond label are similar to the ones from (A).

The variability in *F*_ovS_ increases when approaching the tipping point, and to reduce the variability in the time series, we fit 50 different cubic splines based on 50-year averages (each having a different starting year; see Materials and Methods and fig. S1, C and D). The *F*_ovS_ mean over all the cubic splines is displayed in the inset of Fig. 4A (red curve). Using cubic splines allows us to adequately determine the time derivative of *F*_ovS_ (indicated here by d*_t_F*_ovS_). More interesting is to determine the *F*_ovS_ minimum (i.e., d*_t_F*_ovS_ = 0), because the minimum is an important indicator of the approaching AMOC tipping point. We estimate the *F*_ovS_ minimum using a limited part of the time series before the tipping point. Here, we use at least 100 years of the *F*_ovS_ time series starting from model year 1500, and, for a given period, we simply linearly extrapolate d*_t_F*_ovS_ to find the point in time where d*_t_F*_ovS_ goes through zero. The tipping point estimate fails when analyzing only 100 years of “available” data (model years 1500 to 1600; available data in Fig. 5A). However, extending the available time series by adding “future” data (i.e., future data in Fig. 5A) to the analysis eventually results in a reliable *F*_ovS_ minimum estimate. The result is also robust to the averaging interval when longer than 35 years (Fig. 5B).

**Fig. 6.**
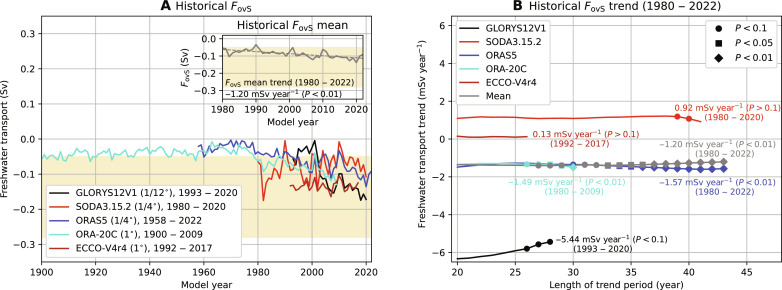
Historical *F*_ovS_ and trend. (**A**) Historical *F*_ovS_ for five different reanalysis and assimilation products. The horizontal resolution for the ocean component and the time span is indicated in the legend. The *F*_ovS_ multi-reanalysis mean (inset) is the yearly average over all available products. The yellow shading indicates observed ranges for *F*_ovS_. (**B**) Present-day (1980 to 2022) *F*_ovS_ trend. We use a sliding window (varying length of 20 to 43 years) over the available time series (1980 to 2022) to determine all *F*_ovS_ trends and then determine the mean trend, which is displayed for each reanalysis product and the multi-reanalysis mean. The markers indicate the least significant [two-sided *t* test (*23*)] trend for a given sliding window, and the trends at the maximum sliding window length (only one trend possible) are also displayed.

The historical *F*_ovS_, derived from reanalysis and assimilation products (Fig. 6A), are consistent in the sign of *F*_ovS_ when comparing those to direct observations (*36*, *42*). The reanalysis product mean shows a robust and significant negative *F*_ovS_ trend (of −1.20 mSv year^−1^) over the past 40 years (Fig. 6B), and its magnitude is close to the projected CMIP6 mean trend [of −1.06 mSv year^−1^, 2000–2100 (*37*)] under a high-end climate change scenario. This multi-reanalysis mean negative trend suggests that the AMOC is on course to tipping as a more negative *F*_ovS_ is associated with a stronger salt-advection feedback. Although the reanalysis products are known to have different biases (*43*), this trend estimate is the best result that can be obtained at the moment. However, these products are too short (maximum of ∼100 years) at the moment to adequately estimate the distance to the *F*_ovS_ minimum.

**Fig. 7.**
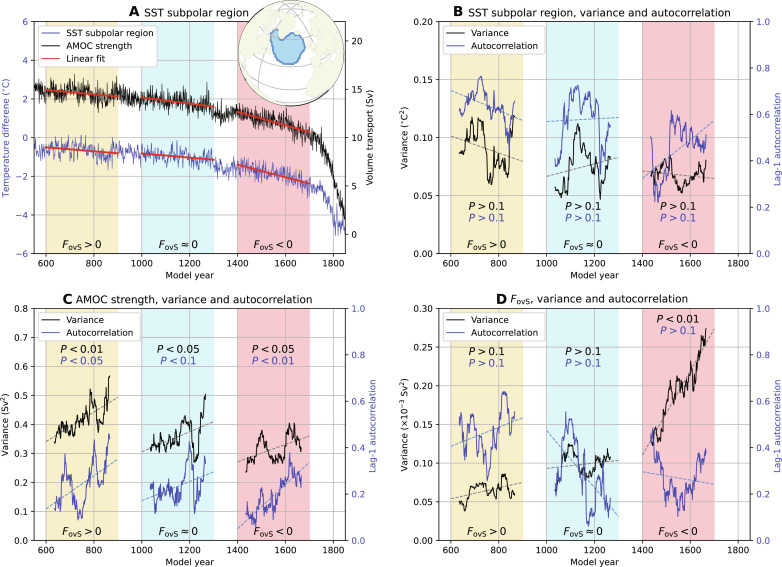
Classical early warning indicators. (**A**) Spatially averaged SST over the subpolar region (blue outlined region in inset) and AMOC strength (similar to Fig. 1A) before the AMOC collapse. The SST time series is averaged over the months November to May, where first the global mean SST [November to May (*4*)] and then the time mean over the first 50 years are subtracted. (**B**) Variance and (lag-1) autocorrelation of the SST subpolar region for three 300-year periods. For each 300-year period, the linear trend is removed [red lines in (A)] before determining the variance and autocorrelation. The variance and autocorrelation are determined over a 70-year sliding window, and the significance of their trends (dashed lines) is indicated for each period [see (*11*) for more details]. (**C** and **D**) Similar to (B) but now for the (C) AMOC strength (A) and (D) *F*_ovS_ (Fig. 4A).

By analyzing SST-based proxies of the AMOC strength (*4*), it was suggested that the real present-day AMOC approaches a tipping point (*11*, *12*). On the basis of idealized models having a saddle-node bifurcation, an increase in both the variance and lag-1 autocorrelation (i.e., the classical early warning indicators) indicates that this bifurcation is approached. Following the same procedure as outlined in (*11*), on our AMOC tipping event in the CESM, we find no consistent increase in the classical early warning indicators for various 300-year periods before the AMOC collapse (Fig. 7, A and B). Shifting and varying the length of the windows analyzed here may eventually result in an increase in both early warning indicators, but over the full time series, these quantities decline when approaching the tipping point and, hence, are not reliable early warning indicators. Another method has been proposed recently to estimate the AMOC tipping point by fitting (and extrapolating) the lag-1 autocorrelation and variance statistics over the 150-year-long (monthly averaged) SST-based AMOC time series (*12*). Following the same procedure as outlined in (*12*), we find an estimate of the tipping point that is consistent with the timing of the *F*_ovS_ minimum. This estimate is only accurate when both the variance and autocorrelation increase (e.g., model years 1427 to 1557, red curves in fig. S7). When shifting the time window, either variance or autocorrelation does not increase (e.g., model years 1503 to 1653, blue curves in fig. S7), resulting in inaccurate tipping point estimates. Because both variance and autocorrelation are increasing in the SST-based AMOC time series in (*12*), their estimate of the tipping point (2025 to 2095, 95% confidence level) could be accurate. On the other hand, our results (fig. S7) also show that the accuracy is sensitive to the time interval analyzed because of decadal variability in the SST time series and that most 150-year time windows do not provide an accurate estimate of the tipping point.

Using the classical early warning indicators directly on the AMOC strength time series (Fig. 7C) gives similar results as the SST time series. The trends over the 300-year periods are significant, but the overall variance and autocorrelation are decreasing toward the tipping point. Only for *F*_ovS_ (Fig. 7D), we find a consistent and significant increase in its variance when approaching the tipping point, and this can also be directly observed from the full-time series (Fig. 4A). The quantity *F*_ovS_, in particular its minimum in combination with its variance increase, is hence a very promising early warning signal for a (future) AMOC collapse.

## DISCUSSION

The results here give a clear answer to a long-standing problem around in the climate research community concerning the existence of AMOC tipping behavior in GCMs (*33*, *44*–*48*). Yes, it does occur in these models. This is bad news for the climate system and humanity as up until now one could think that AMOC tipping was only a theoretical concept and tipping would disappear as soon as the full climate system, with all its additional feedbacks, was considered. On the other hand, the tipping is consistent with the wealth of paleoclimate evidence that rapid changes have occurred in the AMOC, in particular during Dansgaard-Oeschger events (*10*).

The AMOC collapse dramatically changes the redistribution of heat (and salt) and results in a cooling of the Northern Hemisphere, while the Southern Hemisphere slightly warms. Atmospheric and sea-ice feedbacks, which were not considered in idealized climate models studies (*29*, *31*, *32*, *40*), further amplify the AMOC-induced changes, resulting in a very strong and rapid cooling of the European climate with temperature trends of more than 3°C per decade. In comparison with the present-day global mean surface temperature trend (due to climate change) of about 0.2°C per decade, no realistic adaptation measures can deal with such rapid temperature changes under an AMOC collapse (*49*, *50*).

We have developed a physics-based, and observable (*36*, *42*), early warning signal characterizing the tipping point of the AMOC: the minimum of the AMOC-induced freshwater transport at 34°S in the Atlantic, here indicated by *F*_ovS_. The *F*_ovS_ minimum occurs 25 years (9 to 41, 10 and 90% percentiles) before the AMOC tipping event. The quantity *F*_ovS_ has a strong basis in conceptual models, where it is an indicator of the salt-advection feedback strength. Although *F*_ovS_ has been shown to be a useful measure of AMOC stability in GCMs (*51*), the minimum feature has so far not been connected to the tipping point because an AMOC tipping event had up to now not been found in these models. The *F*_ovS_ indicator is observable, and reanalysis products show that its value and, more importantly, its trend are negative at the moment. The latest CMIP6 model simulations indicate that *F*_ovS_ is projected to decrease under future climate change (*37*). However, because of freshwater biases, the CMIP6 *F*_ovS_ mean starts at positive values and only reaches zero around the year 2075 (*37*). Hence, no salt-advection feedback–induced tipping is found yet in these models under climate change scenarios up to 2100 and longer simulations under stronger forcing would be needed (as we do here for the CESM) to find this. In observations, the estimated mean value of *F*_ovS_ is already quite negative, and therefore, any further decrease is in the direction of a tipping point (and a stronger salt-advection feedback). A slowdown in the *F*_ovS_ decline indicates that the AMOC tipping point is near.

In addition, with future observations, an estimate of the distance to the AMOC tipping point can in principle be obtained. Deploying machine learning techniques on *F*_ovS_, in combination with its variance, could also help in estimating the distance to AMOC tipping. We have shown that current reanalysis products provide insufficient information to adequately estimate this distance. Sustained future section measurements (available since 2009) at 34°S from the SAMoc Basin-wide Array (SAMBA) (*52*–*54*) are therefore of utmost importance and will become crucial to estimate the distance to an AMOC collapse. Given the different biases in reanalysis products (*43*) and uncertainties in future climate change, we are currently not able to determine a useful estimate of how many more years would be needed to make a reliable *F*_ovS_ minimum estimate.

In the CESM simulation here, AMOC tipping occurs at relatively large values of the freshwater forcing. This is due to biases in precipitation elsewhere in the models and mainly over the Indian Ocean (*37*). Hence, we needed to integrate the CESM to rather large values of the freshwater forcing [∼0.6 Sv, about a factor 80 times larger than the present-day melt rate of the Greenland Ice Sheet (*55*)] to find the AMOC tipping event. The effect of the biases can be seen from the value of the AMOC-induced freshwater transport at 34°S, *F*_ovS_, which is positive at the start of the simulation. When biases are corrected in the CESM, it is expected that the AMOC tipping is expected to occur at smaller values of the freshwater forcing. As also the present-day background climate state and the climate change forcing are different than in our simulations, the real present-day AMOC may be much closer to its tipping point than in the simulations shown here. Note that the analysis of the early warning signal is not affected by these biases, as this analysis is independent of the background state and precise forcing details.

## MATERIALS AND METHODS

### Climate model simulations

The CESM (in the f19 g16 configuration) is a fully coupled climate model. The Parallel Ocean Program version 2 [POP2; (*56*)] is used for the ocean component, the Community Atmosphere Model version 4 [CAM4; (*57*)] is used for the atmosphere component, and the Community Ice Code version 4 [CICE4; (*58*)] is used for the sea-ice component. The hosing experiment was branched off from the end (model year 2800) of the preindustrial CESM control simulation from Baatsen *et al.* (*16*). Here, it is shown that the upper 1000 m of the ocean is well equilibrated after 2800 years of model integration.

#### The freshwater transport

The total meridional freshwater transport (*F*_∇_) is decomposed into an overturning component (*F*_ov_) and an azonal (gyre) component (*F*_az_), which are determined as

$$F_{\nabla }\left(y\right)=-\frac{1}{S_{0}}\int_{-H}^{0}\int_{x_{W}}^{x_{E}}v\left(S-S_{0}\right)\text{dxdz}$$ (1a)

$$F_{\text{ov}}\left(y\right)=-\frac{1}{S_{0}}\int_{-H}^{0}\left[\int_{x_{W}}^{x_{E}}v^{*}\mathrm{dx}\right]\left[\left\langle S\right\rangle -S_{0}\right]\mathrm{dz}$$ (1b)

$$F_{\text{az}}\left(y\right)=-\frac{1}{S_{0}}\int_{-H}^{0}\int_{x_{W}}^{x_{E}}v\prime S\prime \text{dxdz}$$ (1c)

where *S*_0_ = 35 g kg^−1^ is a reference salinity. *v*^∗^ is defined as *v*^∗^ = *v* − $\hat{v}$ , where *v* is the meridional velocity and $\hat{v}$ is the section spatially averaged meridional velocity. The quantity ⟨*S*⟩ indicates the zonally averaged salinity, and primed quantities (*v*′ and *S*′) are deviations from their respective zonal means. The total freshwater transport also contains a barotropic and eddy (parameterized) component, but these contributions in the 1° ocean resolution CESM setup are very small (*59*) and therefore not included here.

#### The freshwater budget

The freshwater budget over the Atlantic Ocean (34°S to 60°N) is defined as

$$\frac{d\bar{W}}{\mathrm{dt}}=F_{\nabla }^{b}+F_{\text{surf}}+F_{\text{mix}}$$ (2a)

$$\bar{W}=-\frac{1}{S_{0}}\int_{-H}^{0}\int_{34^{\circ }S}^{60^{\circ }N}\int_{x_{W}}^{x_{E}}\left(S-S_{0}\right)\text{dxdydz}$$ (2b)

where $\bar{W}$ is the freshwater content, $F_{\nabla }^{b}$ is the freshwater convergence and is determined as the freshwater transport through the three boundaries (i.e., 34°S, 60°N, and the Strait of Gibraltar), *F*_surf_ is the surface freshwater flux, and *F*_mix_ is a residual term that closes the budget and captures, for example, diffusion (*59*). The Atlantic’s surface freshwater flux is primarily dominated by precipitation and evaporation but also includes runoff (river and land ice), sea-ice processes (melt and brine rejection), and the anomalous freshwater forcing (*F*_H_).

#### The AMOC strength

The AMOC strength is defined as the total meridional volume transport at 26°N over the upper 1000 m

$$\text{AMOC}\left(y=26^{\circ }N\right)=\int_{-1000}^{0}\int_{x_{W}}^{x_{E}}v\;\text{dxdz}$$ (3)

#### F_ovS_ minimum estimate

To estimate the *F*_ovS_ minimum, we use cubic splines that interpolate piece-wise, between so-called knots, cubic polynomials that are twice continuously differentiable, and we impose that the second derivate is zero at the first and last knot. The knots are determined over *n*-year averages of the *F*_ovS_ time series for different starting years (1, 2, ..., *n* − 1; fig. S1C) and result in *n* different cubic splines (fig. S1D) and their respective derivatives (d*_t_F*_ovS_). Using a linear fit over the cubic spline mean derivative, we estimate where the derivative goes through zero (i.e., the *F*_ovS_ minimum; Fig. 5). A minimum of *n* ≥ 35 (year averages and cubic splines) is required to substantially reduce the variability of the time series and find a consistent *F*_ovS_ minimum estimate.

### Software and model output

The (processed) model output and analysis scripts are provided at: https://doi.org/10.5281/zenodo.10461549. The reanalysis and assimilation products can be accessed through GLORYS12V1 (https://doi.org/10.48670/moi-00021), SODA3.15.2 (http://soda.umd.edu), ORAS5 (https://doi.org/10.24381/cds.67e8eeb7), ORA-20C (https://icdc.cen.uni-hamburg.de/thredds/catalog/ftpthredds/EASYInit/ora20c/opa0/catalog.html), and ECCO-V4r4 (https://ecco-group.org/products-ECCO-V4r4.htm).

## Supplementary Materials

### This PDF file includes:

Figs. S1 to S7 References
