# Supplementary material for: Physics-based early warning signal shows that AMOC is on tipping course
Source: Sci Adv. 2024 Feb 9;10(6):eadk1189. doi: 10.1126/sciadv.adk1189 (PMC10857529; doi:10.1126/sciadv.adk1189)
Supplement: Supplementary file 1 — Figs. S1 to S7 References [file sciadv.adk1189_sm.pdf]

Science Advances
AAAS

Supplementary Materials for

Physics-based early warning signal shows that AMOC is on tipping course

René M. van Westen *et al.*

Corresponding author: René M. van Westen, r.m.vanwesten@uu.nl

Sci. Adv. **10**, eadk1189 (2024)
DOI: 10.1126/sciadv.adk1189

This PDF file includes:

Figs. S1 to S7
References

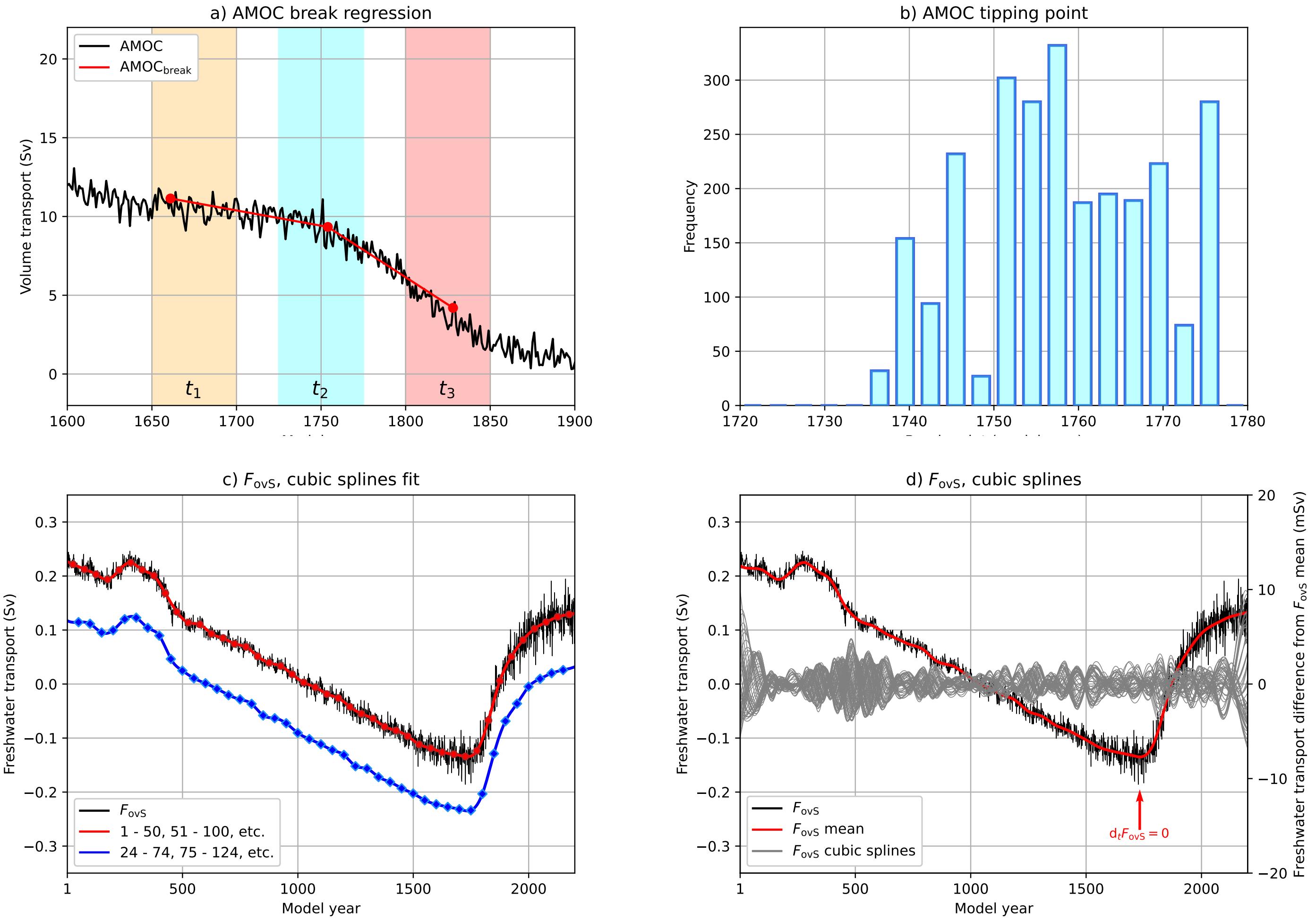

Figure S1: AMOC tipping point and F_{ovS} minimum (a): Break regression analysis (*39*) on the AMOC strength, indicated by the red curve (AMOC$_{\mathrm{break}}$). The change in the linear trend between the two periods (t_1 to t_2 and t_2 to t_3) indicates the AMOC tipping point. The tipping point is found when minimising the quantity S = (AMOC - AMOC$_{\mathrm{break}}$)^2 for varying t_2 (model years 1,725 – 1,775), the linear trends must be negative between both periods. (b): The AMOC tipping point for varying t_1 (model years 1,650 – 1,700) and varying t_3 (model years 1,800 – 1,850), the mean is at model year 1,758. (c): Cubic spline interpolation of F_{ovS}, where the knots are 50-year averages of the F_{ovS} for different starting years (red = model year 1, blue = model year 25). The blue time series is shifted vertically for visibility reasons. (d): The F_{ovS} mean over all the 50 different cubic splines, the cubic splines are displayed as deviations from the F_{ovS} mean. The F_{ovS} (mean) minimum (i.e., $\mathrm{d}_t F_{\mathrm{ovS}} = 0$) is at model year 1,732

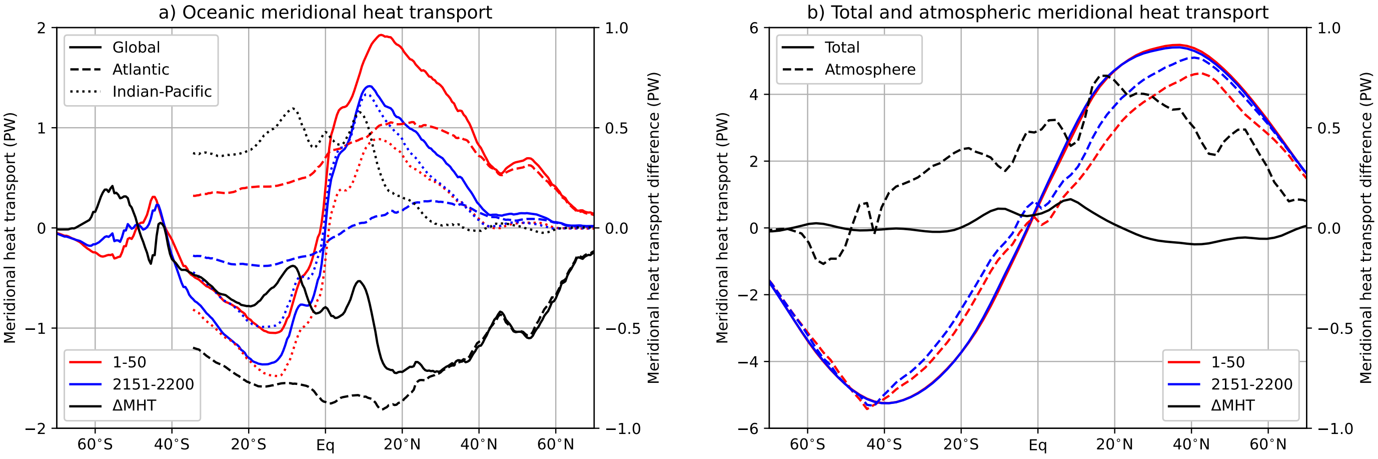

Figure S2: Meridional heat transport (a & b): The total, oceanic and atmospheric meridional heat transport (MHT) for model years 1 – 50 (red curves) and 2,151 – 2,200 (blue curves) and differences (black curves) between the two periods. In panel a the meridional heat transport is shown for the global ocean (solid curves), Atlantic Ocean (dashed curves) and Indian-Pacific Ocean (dotted curves). In panel b the global meridional heat transport is displayed. Note the different vertical ranges between the two panels.

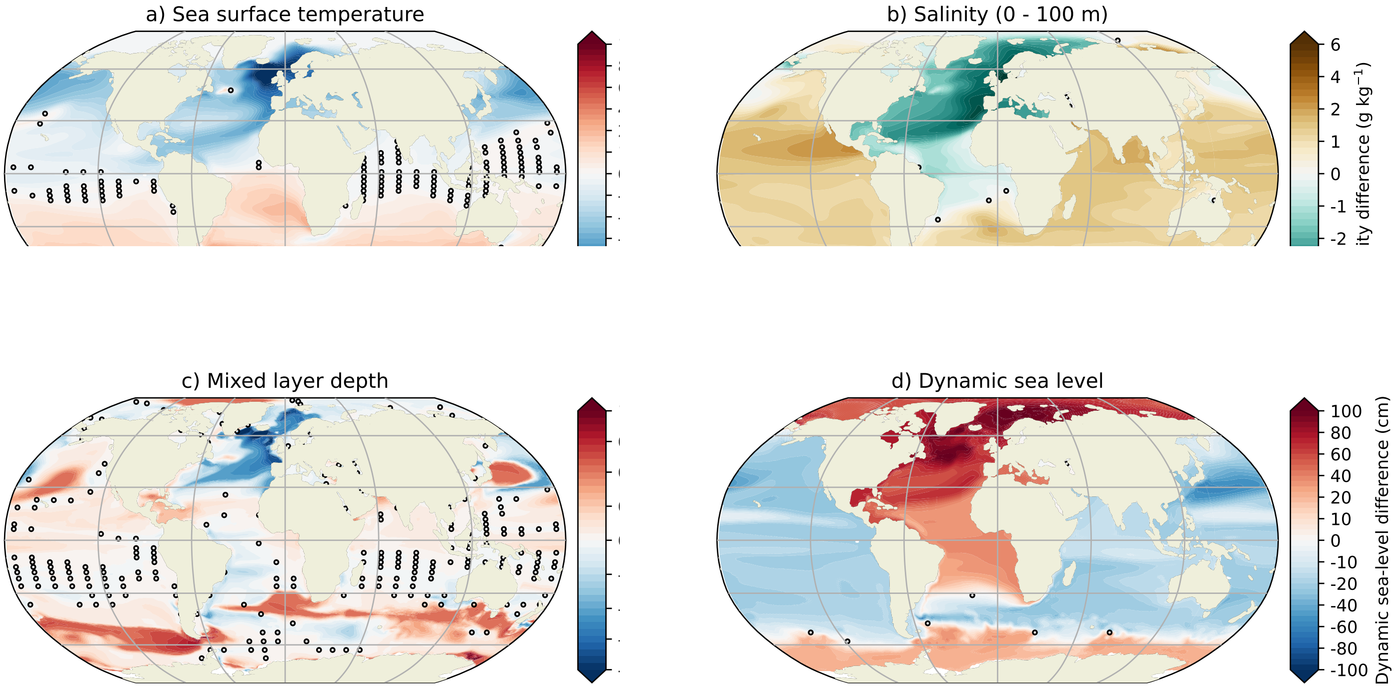

Figure S3: Oceanic response. (a): The sea surface temperature differences between the two AMOC states (model years 2,151 – 2,200 minus 1 – 50), the markers indicate non-significant ($p \geq 0.05$, two-sided Welch's t-test) differences. (b – d): Similar to panel a, but now for the (b): vertically-averaged (0 – 100 m) salinity, (c): yearly-maximum mixed layer depth and (d): dynamic sea level.

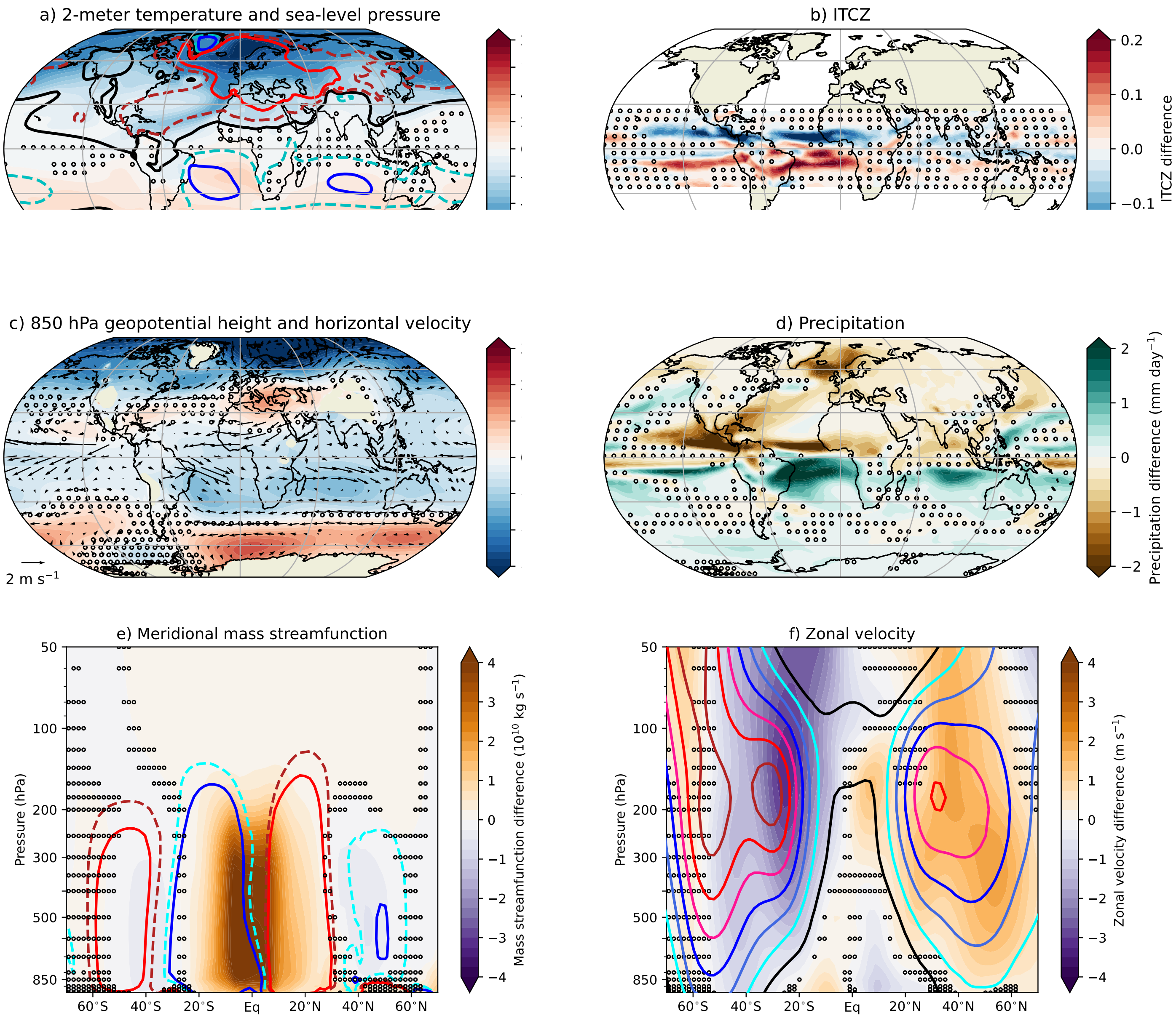

Figure S4: Atmospheric response. (a): The 2-meter surface temperature differences between the two AMOC states (model years 2,151 – 2,200 minus 1 – 50), the markers indicate non-significant ($p \geq 0.05$, two-sided Welch's t-test) differences. The red (blue) curves show positive (negative) values of sea-level pressure differences with magnitudes of (-)1 hPa and (-)2 hPa for the dashed and solid curves, respectively. (b – f): Similar to panel a, but now for the (b): ITCZ location probability density function, (c): 850 hPa geopotential height (shading) and 850 hPa horizontal velocities (quivers), (d): precipitation. (e): meridional mass streamfunction and (f): zonally-averaged zonal velocity. The ITCZ location is determined from the monthly-averaged joint distribution of outgoing longwave radiation and precipitation and are then converted to yearly probabilities (*62*). The curves in panel e show the meridional mass streamfunction for model years 1 – 50, where the red (blue) curves are positive (negative) value with magnitudes of $(-)1 \times 10^{10}$ kg s^{-1} and $(-)2 \times 10^{10}$ kg s^{-1} for the dashed and solid curves, respectively. The curves in panel f show the zonal velocity for model years 1 – 50, where the black curve is 0 m s^{-1} and the coloured curves are spaced every +5 m s^{-1}.

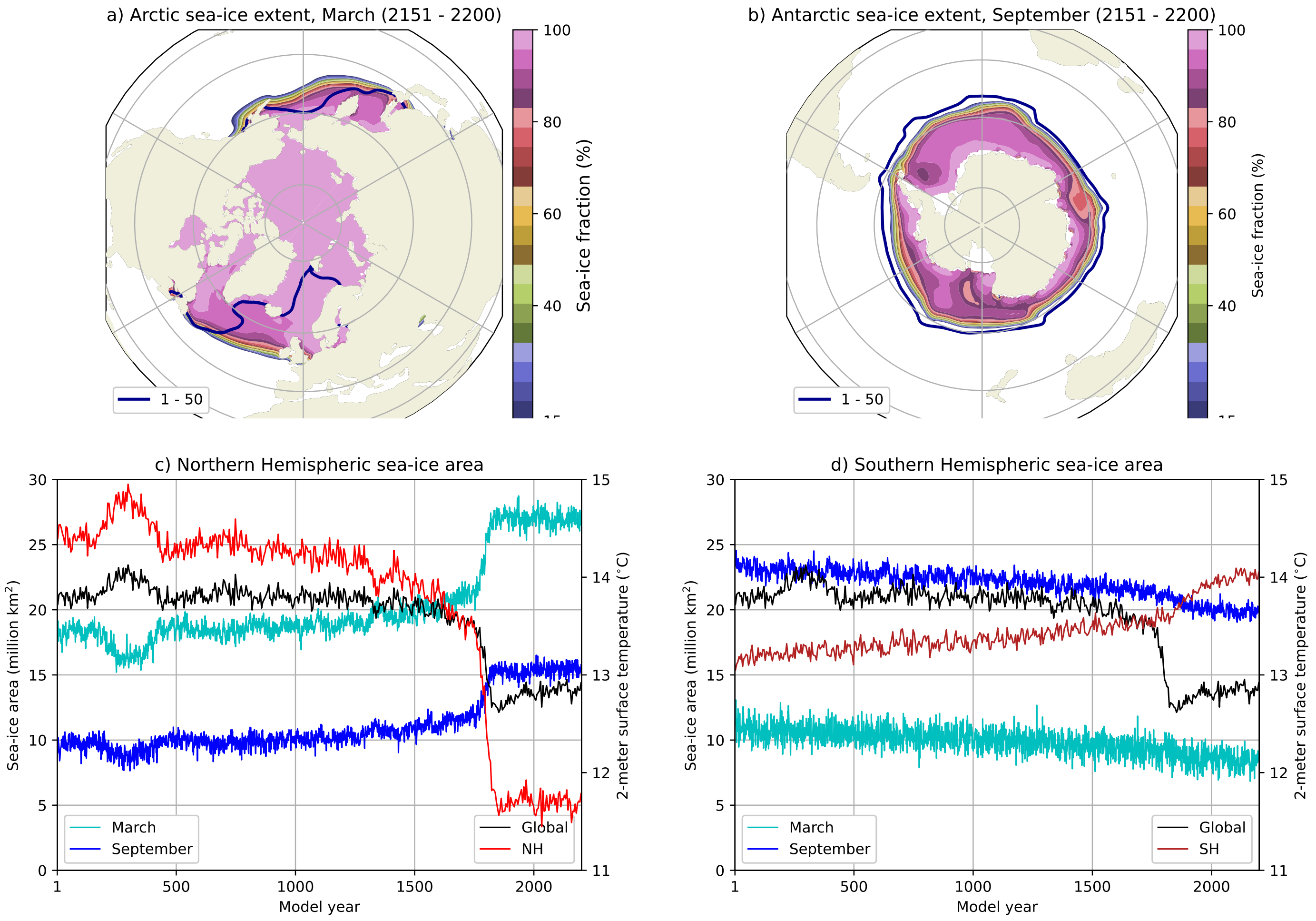

Figure S5: Sea-ice response. (a): The Arctic sea-ice fraction for March for model years 2,151 – 2,200. The dark blue curve shows the Arctic sea-ice edge (i.e, the 15% sea-ice fraction isoline) for March for model years 1 – 50. (b): Similar to panel a, but now for the Antarctic sea-ice fractions for September. (c): The Northern Hemispheric sea-ice area for March and September, including the 2-meter surface temperature for the global mean and Northern Hemisphere. The sea-ice area is based on all the grid cells with sea-ice fractions larger than 15%. The 2-meter surface temperature time series are displayed as 5-year averages (to reduce the variability of the time series). (d): Similar to panel c, but now for the Southern Hemispheric sea-ice area and the Southern Hemispheric 2-meter surface temperature.

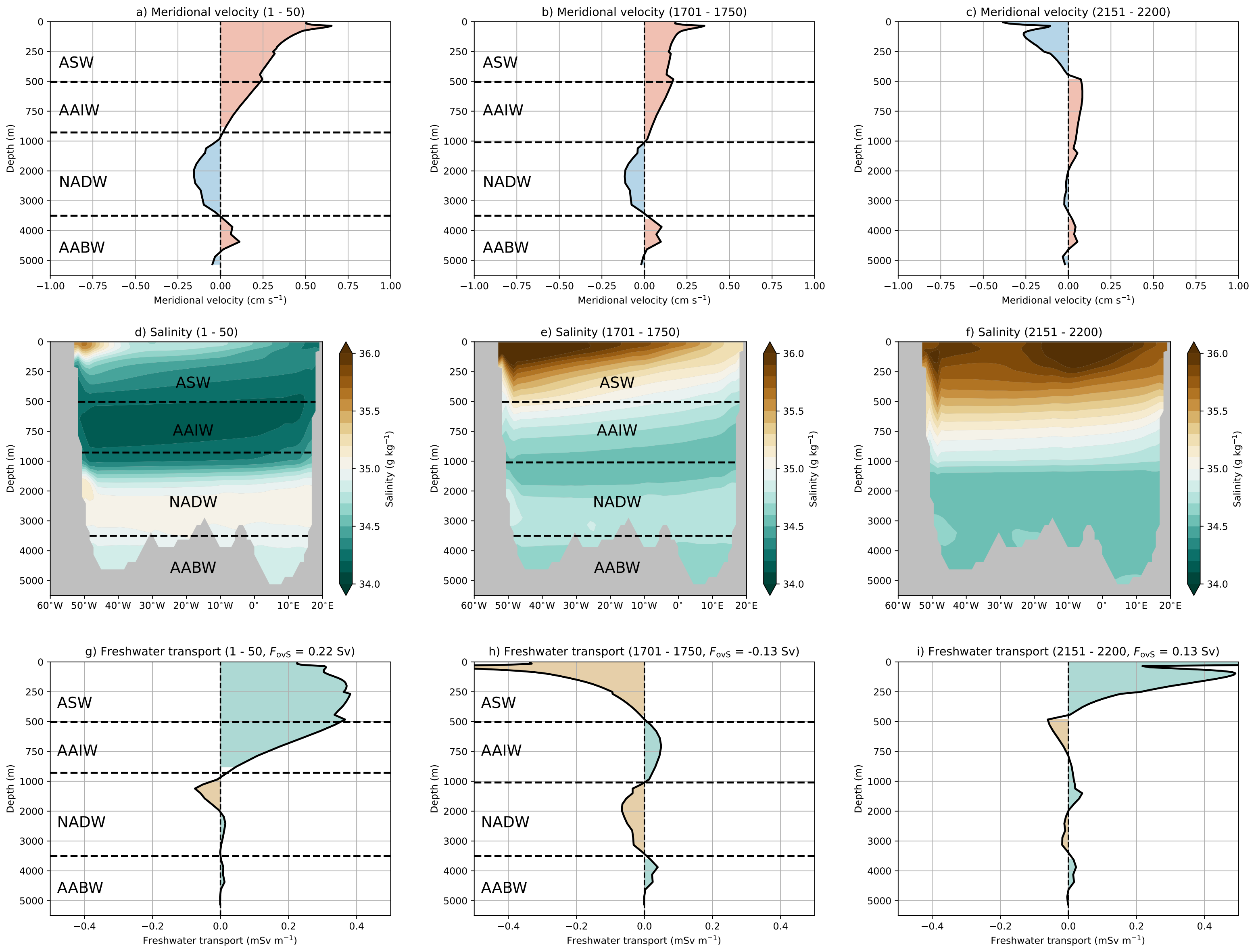

Figure S6: Water mass properties at 34°S. (Upper row): The zonally-averaged meridional velocity at 34°S for three periods (model years 1 – 50, 1,701 – 1,750 and 2,151 – 2,200). (Middle row): The salinity along 34°S for the three periods. (Lower row): The freshwater transport (overturning component) with depth at 34°S for the three periods. The different water masses are derived from the velocity profile (*37*) and is only applicable for the northward overturning circulation (left and middle column) and the names are: Atlantic Surface Water (ASW), Antarctic Intermediate Water (AAIW), North Atlantic Deep Water (NADW) and Antarctic Bottom Water (AABW).

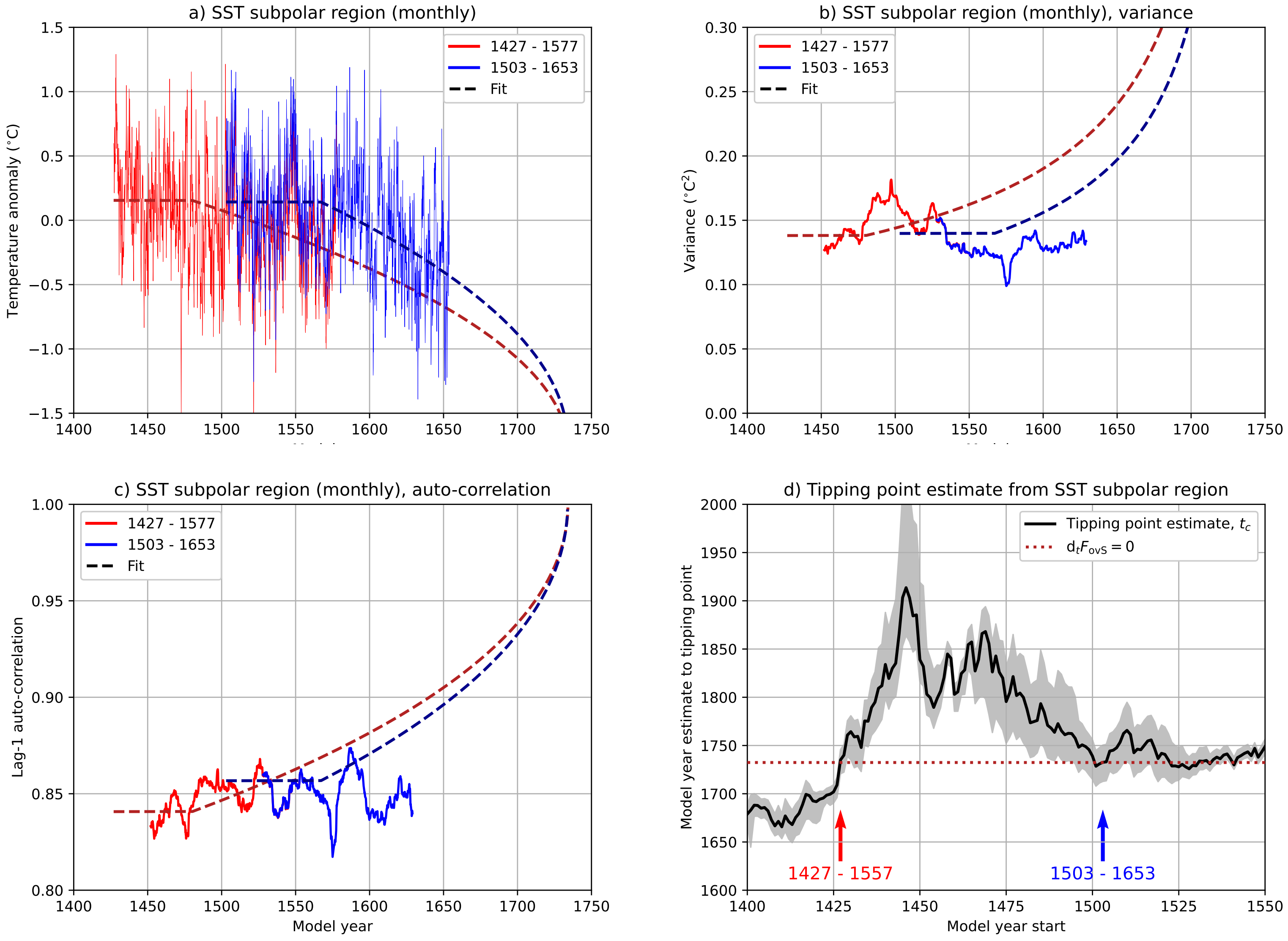

Figure S7: Tipping point estimate from SST subpolar time series. (a): The monthly SST subpolar time series (solid curves) for two 150-year periods. For each 150-year period, the monthly SST over the subpolar region (blue outlined region in inset in Figure 7a) and the monthly globally-averaged SST are determined. Next, the monthly mean (i.e., January months, February months, etc.) is removed for both time series and then the globally-averaged SST anomaly time series is subtracted from the SST subpolar region anomaly time series. We did not subtract twice the globally-averaged SST (*12*) as there is no climate change scenario in the CESM. (b & c): The variance and lag-1 auto-correlation (solid curves) of the monthly SST subpolar time series over a 50-year sliding window. A linear trend is removed over each sliding window before determining the variance and auto-correlation. (d): The tipping point estimate, t_c, from the procedure outlined in (*12*) over 150-year periods and varying starting year. An example of the tipping point estimate (auto-correlation approaches 1) is shown by the dashed curves in panels a – c. The shading indicates the 95%-confidence interval and is determined by varying the length of the stationary part (50 to 80 years).

REFERENCES AND NOTES

1. W. E. Johns, M. O. Baringer, L. M. Beal, S. A. Cunningham, T. Kanzow, H. L. Bryden, J. J. M. Hirschi, J. Marotzke, C. S. Meinen, B. Shaw, R. Curry, Continuous, array-based estimates of Atlantic Ocean heat transport at 26.5 N. J. Climate 24, 2429–2449 (2011).

2. M. A. Srokosz, H. L. Bryden, Observing the Atlantic meridional overturning circulation yields a decade of inevitable surprises. Science 348, 1255575 (2015).

3. I . Moat Ben, A. Smeed David, F.-W. Eleanor, D. Desbruyères, B. Claudie, E. J. William, R. Darren, S.-F. Alejandra, O. B. Molly, V. Denis, C. J. Laura, L. B. Harry, Pending recovery in the strength of the meridional overturning circulation at 26° N. Ocean Sci. 16, 863–874 (2020).

4. L . Caesar, S. Rahmstorf, A. Robinson, G. Feulner, V. Saba, Observed fingerprint of a weakening Atlantic Ocean overturning circulation. Nature 556, 191–196 (2018).

5. L . Caesar, G. D. McCarthy, D. J. R. Thornalley, N. Cahill, S. Rahmstorf, Current Atlantic meridional overturning circulation weakest in last millennium. Nat. Geosci. 14, 118–120 (2021).

6. T . M. Lenton, H. Held, E. Kriegler, J. W. Hall, W. Lucht, S. Rahmstorf, H. J. Schellnhuber, Tipping elements in the Earth's climate system. Proc. Natl. Acad. Sci. U. S. A. 105, 1786–1793 (2008).

7. D . I. Armstrong McKay, A. Staal, J. F. Abrams, R. Winkelmann, B. Sakschewski, S. Loriani, I. Fetzer, S. E. Cornell, J. Rockström, T. M. Lenton, Exceeding 1.5 C global warming could trigger multiple climate tipping points. Science 377, eabn7950 (2022).

8. S. Rahmstorf, Ocean circulation and climate during the past 120,000 years. Nature 419, 207–214 (2002).

9. L . G. Henry, J. F. McManus, W. B. Curry, N. L. Roberts, A. M. Piotrowski, L. D. Keigwin, North Atlantic ocean circulation and abrupt climate change during the last glaciation. Science

353, 470–474 (2016).

10. J. Lynch-Stieglitz, The Atlantic meridional overturning circulation and abrupt climate change. Ann. Rev. Mar. Sci. 9, 83–104 (2016).

11. N . Boers, Observation-based early-warning signals for a collapse of the Atlantic meridional overturning circulation. Nat. Clim. Change 11, 680–688 (2021).

12. P. Ditlevsen, S. Ditlevsen, Warning of a forthcoming collapse of the Atlantic meridional overturning circulation. Nat. Commun. 14, 4254 (2023).

13. P. D. Ditlevsen, S. J. Johnsen, Tipping points: Early warning and wishful thinking. Geophys. Res. Lett. 37, L19703 (2010).

14. C . Kuehn, A mathematical framework for critical transitions: Bifurcations, fast–slow systems and stochastic dynamics. Phys. D Nonlinear Phenom. 240, 1020–1035 (2011).

15. S. Qin, C. Tang, Early-warning signals of critical transition: Effect of extrinsic noise. Phys. Rev. E 97, 032406 (2018).

16. M. Baatsen, A. S. Von Der Heydt, M. Huber, M. A. Kliphuis, P. K. Bijl, A. Sluijs, H. A. Dijkstra, The middle to late Eocene greenhouse climate modelled using the CESM 1.0.5. Clim. Past 16, 2573–2597 (2020).

17. S. Rahmstorf, M. Crucifix, A. Ganopolski, H. Goosse, I. Kamenkovich, R. Knutti, G. Lohmann, R. Marsh, L. A. Mysak, Z. Wang, A. J. Weaver, Thermohaline circulation hysteresis: A model intercomparison. Geophys. Res. Lett. 32, L23605 (2005).

18. E . Hawkins, R. S. Smith, L. C. Allison, J. M. Gregory, T. J. Woollings, H. Pohlmann, B. de Cuevas, Bistability of the Atlantic overturning circulation in a global climate model and links to ocean fresh-water transport. Geophys. Res. Lett. 38, L10605 (2011).

19. A. Hu, G. A. Meehl, W. Han, A. Timmermann, B. Otto-Bliesner, Z. Liu, W. M. Washington, W. Large, A. Abe-Ouchi, M. Kimoto, K. Lambeck, B. Wu, Role of the Bering Strait on the hysteresis of the ocean conveyor belt circulation and glacial climate stability. Proc. Natl. Acad. Sci. U. S. A. 109, 6417 6422 (2012).

20. B. Orihuela-Pinto, M. H. England, A. S. Taschetto, Interbasin and interhemispheric impacts of a collapsed Atlantic overturning circulation. Nat. Clim. Change 12, 558–565 (2022).

21. J. Mecking, S. Drijfhout, L. Jackson, M. Andrews, The effect of model bias on Atlantic freshwater transport and implications for AMOC bi-stability. Tellus A Dyn. Meteorol. Oceanogr. 69, 1299910 (2022).

22. T . F. Stocker, The seesaw effect. Science 282, 61–62 (1998).

23. B. D. Santer, T. M. L. Wigley, J. S. Boyle, D. J. Gaffen, J. J. Hnilo, D. Nychka, D. E. Parker, K. E. Taylor, Statistical significance of trends and trend differences in layer-average atmospheric temperature time series. J. Geophys. Res. Atmos. 105, 7337–7356 (2000).

24. M. Hirota, M. Holmgren, E. H. Van Nes, M. Scheffer, Global resilience of tropical forest and savanna to critical transitions. Science 334, 232–235 (2011).

25. N . Boers, N. Marwan, H. M. Barbosa, J. Kurths, A deforestation-induced tipping point for the South American monsoon system. Sci. Rep. 7, 41489 (2017).

26. M. M. Dekker, A. S. Von Der Heydt, H. A. Dijkstra, Cascading transitions in the climate system. Earth System Dyn. Dynamics 9, 1243–1260 (2018).

27. N . Wunderling, J. F. Donges, J. Kurths, R. Winkelmann, Interacting tipping elements increase risk of climate domino effects under global warming. Earth Syst. Dynam. 12, 601–619 (2021).

28. A. K. Klose, N. Wunderling, R. Winkelmann, J. F. Donges, What do we mean, ‘tipping cascade’? Environ. Res. Lett. 16, 125011 (2021).

29. S. Rahmstorf, On the freshwater forcing and transport of the Atlantic thermohaline circulation. Clim. Dyn. 12, 799–811 (1996).

30. P. de Vries, S. L. Weber, The Atlantic freshwater budget as a diagnostic for the existence of a stable shut down of the meridional overturning circulation. Geophys. Res. Lett. 32, 2004GL021450 (2005).

31. H . A. Dijkstra, Characterization of the multiple equilibria regime in a global ocean model. Tellus A Dyn. Meteorol. Oceanogr. 59, 695–705 (2022).

32. S. E. Huisman, M. Den Toom, H. A. Dijkstra, S. Drijfhout, An indicator of the multiple equilibria regime of the Atlantic meridional overturning circulation. J. Phys. Oceanogr. 40, 551–567 (2010).

33. W. Weijer, W. Cheng, S. S. Drijfhout, A. V. Fedorov, A. Hu, L. C. Jackson, W. Liu, E. L. McDonagh, J. V. Mecking, J. Zhang, Stability of the Atlantic meridional overturning circulation: A review and synthesis. J. Geophys. Res. Oceans 124, 5336–5375 (2019).

34. J. Marotzke, Abrupt climate change and thermohaline circulation: Mechanisms and predictability. Proc. Natl. Acad. Sci. U. S. A. 97, 1347–1350 (2000).

35. W. R. Peltier, G. Vettoretti, Dansgaard-Oeschger oscillations predicted in a comprehensive model of glacial climate: A “kicked” salt oscillator in the Atlantic. Geophys. Res. Lett. 41, 7306–7313 (2014).

36. H . L. Bryden, B. A. King, G. D. McCarthy, South Atlantic overturning circulation at 24 S. J. Mar. Res. 69, 38–55 (2011).

37. R. M. van Westen, H. A. Dijkstra, Persistent climate model biases in the Atlantic Ocean’s freshwater transport. EGUsphere, 1–29 (2023).

38. S. S. Drijfhout, S. L. Weber, E. van der Swaluw, The stability of the MOC as diagnosed from model projections for pre-industrial, present and future climates. Clim. Dyn. 37, 1575–1586 (2011).

39. M. Mudelsee, T. Bickert, C. H. Lear, G. Lohmann, Cenozoic climate changes: A review based on time

series analysis of marine benthic δ18O records. Rev. Geophys. 52, 333–374 (2014).

40. P. Cessi, A simple box model of stochastically forced thermohaline flow. J. Phys. Oceanogr. 24, 1911–1920 (1994).

41. H . Stommel, Thermohaline convection with two stable regimes of flow. Tellus 13, 224–230 (1961).

42. S. L. Garzoli, M. O. Baringer, S. Dong, R. C. Perez, Q. Yao, South Atlantic meridional fluxes. Deep-Sea Res. I Oceanogr. Res. Pap. 71, 21–32 (2013).

43. L . C. Jackson, A. Biastoch, M. W. Buckley, D. G. Desbruyères, E. Frajka-Williams, B. Moat, J. Robson, The evolution of the North Atlantic meridional overturning circulation since 1980. Nat. Rev. Earth Environ. 3, 241–254 (2022).

44. R. J. Stouffer, J. Yin, J. M. Gregory, K. W. Dixon, M. J. Spelman, W. Hurlin, A. J. Weaver, M. Eby, G. M. Flato, H. Hasumi, A. Hu, J. H. Jungclaus, I. V. Kamenkovich, A. Levermann, M. Montoya, S. Murakami, S. Nawrath, A. Oka, W. R. Peltier, D. Y. Robitaille, A. Sokolov, G. Vettoretti, S. L. Weber, Investigating the causes of the response of the thermohaline circulation to past and future climate changes. J. Climate 19, 1365–1387 (2006).

45. J. Mecking, S. S. Drijfhout, L. C. Jackson, T. Graham, Stable AMOC off state in an eddy-permitting coupled climate model. Clim. Dyn. 47, 2455–2470 (2016).

46. L . C. Jackson, R. A. Wood, Timescales of AMOC decline in response to fresh water forcing. Clim. Dyn. 51, 1333–1350 (2018).

47. W. Liu, Z. Liu, E. C. Brady, Why is the AMOC monostable in coupled general circulation models? J. Climate 27, 2427–2443 (2014).

48. L . Jackson, R. Wood, Hysteresis and resilience of the AMOC in an Eddy-permitting GCM. Geophy. Res. Lett. 45, 8547–8556 (2018).

49. C . Vogel, K. O’Brien, Vulnerability and global environmental change: Rhetoric and reality.

Aviso (2004).

50. J. Birkmann, M. Garschagen, F. Kraas, N. Quang, Adaptive urban governance: New challenges for the second generation of urban adaptation strategies to climate change. Sustain. Sci. 5, 185–206 (2010).

51. L . C. Jackson, Shutdown and recovery of the AMOC in a coupled global climate model: The role of the advective feedback. Geophys. Res. Lett. 40, 1182–1188 (2013).

52. C . S. Meinen, S. Speich, A. R. Piola, I. Ansorge, E. Campos, M. Kersalé, T. Terre, M. P. Chidichimo, T. Lamont, O. T. Sato, R. C. Perez, D. Valla, M. van den Berg, M. L. Hénaff, S. Dong, S. L. Garzoli, Meridional overturning circulation transport variability at 34.5°S During 2009–2017: Baroclinic and barotropic flows and the dueling influence of the boundaries. Geophys. Res. Lett. 45, 4180–4188 (2018).

53. M. Kersalé, C. S. Meinen, R. C. Perez, M. Le Hénaff, D. Valla, T. Lamont, O. T. Sato, S. Dong, T. Terre, M. van Caspel, M. P. Chidichimo, M. van den Berg, S. Speich, A. R. Piola, E. J. D. Campos, I. Ansorge, D. L. Volkov, R. Lumpkin, S. L. Garzoli, Highly variable upper and abyssal overturning cells in the South Atlantic. Sci. Adv. 6, eaba7573 (2020).

54. M. Kersalé, C. S. Meinen, R. C. Perez, A. R. Piola, S. Speich, E. J. D. Campos, S. L. Garzoli, I. Ansorge, D. L. Volkov, M. Le Hénaff, S. Dong, T. Lamont, O. T. Sato, M. van den Berg, Multi-year estimates of daily heat transport by the Atlantic meridional overturning circulation at 34.5°S. J. Geophys. Res. Oceans 126, e2020JC016947 (2021).

55. I . Sasgen, B. Wouters, A. S. Gardner, M. D. King, M. Tedesco, F. W. Landerer, C. Dahle, H. Save, X. Fettweis, Return to rapid ice loss in Greenland and record loss in 2019 detected by the GRACE-FO satellites. Commun. Earth Environ. 1, 1–8 (2020).

56. R. Smith, P. Jones, B. Briegleb, F. Bryan, G. Danabasoglu, J. Dennis, J. Dukowicz, C. Eden, B. Fox-Kemper, P. Gent, M. Hecht, S. Jayne, M. Jochum, W. Large, K. Lindsay, M. Maltrud, N. Norton, S. Peacock, M. Vertenstein, S. Yeager, The parallel ocean program (POP)

reference manual (Technical Report, 2010).

57. R. B. Neale, J. Richter, S. Park, P. H. Lauritzen, S. J. Vavrus, P. J. Rasch, M. Zhang, The mean climate of the Community Atmosphere Model (CAM4) in forced SST and fully coupled experiments. J. Climate 26, 5150–5168 (2013).

58. E . Hunke, W. Lipscomb, The Los Alamos sea ice model, documentation and software (Technical Report LA-CC-06-012,2008).

59. A. Jüling, Z. Xun, D. Castellana, V. D. Heydt, S. Anna, D. A. Henk, The Atlantic's freshwater budget under climate change in the Community Earth System Model with strongly eddying oceans. Ocean Sci. 17, 729–754 (2021).

60. D . A. Smeed, S. A. Josey, C. Beaulieu, W. E. Johns, B. I. Moat, E. Frajka-Williams, D. Rayner, C. S. Meinen, M. O. Baringer, H. L. Bryden, G. D. McCarthy, The North Atlantic Ocean is in a state of reduced overturning. Geophys. Res. Lett. 45, 1527–1533 (2018).

61. E . L. Worthington, B. I. Moat, D. A. Smeed, J. V. Mecking, R. Marsh, G. D. McCarthy, A 30-year reconstruction of the Atlantic meridional overturning circulation shows no decline. Ocean Sci. 17, 285–299 (2021).

62. A. Mamalakis, J. T. Randerson, J.-Y.Yu, M. S. Pritchard, G. Magnusdottir, P. Smyth, P. A. Levine, S. Yu, E. Foufoula-Georgiou, Zonally contrasting shifts of the tropical rain belt in response to climate change. Nat. Clim. Change 11, 143–151 (2021).
